# The association between self-presentation on social network sites and interpersonal relationship satisfaction among high school students: the chain mediating role of self-esteem and self-identity

**DOI:** 10.3389/fpsyg.2025.1647474

**Published:** 2025-12-04

**Authors:** Wangyang Peng, Genghu Shi, Xiangen Hu, Hongjuan Zhuang

**Affiliations:** 1 Xiamen Academy of Educational Sciences, Xiamen, China; 2 School of Psychology, Central China Normal University, Wuhan, China; 3 Department of Psychology, Faculty of Education, Guangxi Normal University, Guilin, China; 4 Academy for Interdisciplinary Research, The Hong Kong Polytechnic University, Hongkong, China; 5 Xiamen Shuangshi Middle School of Fujian, Xiamen, China

**Keywords:** self-presentation, social network sites, interpersonal relationship satisfaction, self-esteem, self-identity, high school students

## Abstract

**Introduction:**

The development of social networks has reshaped adolescent self-presentation. The mode of self- presentation not only reflects their judgment of self-worth but also molds their self-awareness and interactions. By shaping their understanding and construction of the self, it ultimately affects their perception and evaluation of interpersonal relationships. This research aimed to examine the effect of self-presentation in SNS (Social Network Site) on high school students’ interpersonal satisfaction as well as the mediation effect of self-esteem and self-identity.

**Methods:**

A sample of 711 high school students (47.53% males, mean age = 15.76 years, SD = 0.55) from Xiamen were included in this study. The Questionnaire of Self-presentation in SNS, the Self-Esteem Scale, the Questionnaire of Self-identity and the Questionnaire of Interpersonal Satisfaction were used to collect data. The bootstrap method was used to analyze the mediated effects.

**Results:**

(1) There are significant positive correlations among honest self-presentation in social network sites, self-esteem, self-identity, and interpersonal relationship satisfaction in high school students. (2) Both positive and honest self-presentation in social network sites positively predicted high school students’ interpersonal satisfaction; (3) High school students’ self-esteem and self-identity mediated the relationship between honest self-presentation and interpersonal satisfaction; (4) Self-presentation in online social network sites affected high school students’ interpersonal satisfaction through the chain mediating role of both self-esteem and self-identity.

**Conclusion:**

This study proposes that educators should focus on cultivating high school students’ self-esteem and self-identity. By enhancing their level of self-esteem and facilitating the transition from their actual to ideal self, students can develop greater self-awareness and a stronger sense of self-worth. This process promotes positive interpersonal experiences and ultimately enhances their overall well-being.

## 1 Introduction

According to the 52nd Statistical Report on Internet Development in China released by the China Internet Network Information Center (CNNIC) (2023), the number of internet users in China reached 1.079 billion by June 2023, with adolescents aged 10–19 accounting for 13.9% of the total. In the United States, 95% of teenagers have a smartphone and use it for online social activities, while 45% report being online almost every day. Additionally, 90% of teenagers aged 13 to 17 indicate that they use at least one social media platform (Anderson and Jiang, 2018). The widespread adoption and rapid development of social media platforms have transformed the ways in which adolescents present themselves. At the same time, features such as anonymity, interactivity, and instant feedback on social network sites may also alter adolescents’ motivations for self-presentation on these platforms.

Tedeschi et al. (1971) proposed that individuals’ self-presentation is driven by two types of motivations: one is to meet theirs expectations of social interactions or to facilitate the development of new situational norms; the other one is to gain theirs favorable evaluations or rewards. Hollenbaugh (2021) suggests that the anonymity in online social media can moderate both the motivation and content of self-presentation. For instance, increased anonymity may facilitate self-presentations that are more aligned with an ideal self. This anonymity may also enable users to present identities online that are distinct from their real-life personas. Adolescents may publish exaggerated personal information or butter their audiences on social platforms with the aim of pursue positive feedback, such as, monetary rewards, and other resources. According to Erikson’s theory of psychosocial development, adolescence is a stage characterized by the risk of role confusion and the pursuit of identity formation. During this period, adolescents develop a deeper understanding of themselves through others’ attitudes and the social roles they enact, striving to overcome dependency on parents and establish close friendships with peers. If adolescents successfully accomplish the developmental tasks of this stage, they can achieve self-identity, which is a sense of consistency between how they are perceived by others and their self-awareness, along with the perception that their goals and the means to achieve them are socially recognized (Yu and Luo, 2016). Similarly, Jones and Pittman (1982) suggested that the goal of self-presentation is to appeal to others in order to secure more beneficial resources, reduce the likelihood of being harmed by others, or enhance self-worth. According to this theory, how adolescents express themselves on social network sites not only reflects their judgment of self-worth, self-perception, modes of interacting with others but also how they interpret and integrate feedback from others, that can reshape their self-understanding and construction, as well as their perception and evaluation of interpersonal relationships.

The self-identity development of adolescence is a crucial period for social and attribute development. It is noteworthy that self-presentation on social media tends to be more virtual than in the real world. Adolescents may receive positive feedback by presenting an idealized self-image online, which can contribute to more positive interpretations of interpersonal relationships and higher satisfaction. However, the anonymity of the internet means that feedback on adolescents’ self-presentation may not always be objective or truthful, and may even include malicious or negative comments. Such interactions could potentially lead to negative outcomes, including unfavorable self-evaluations and pessimistic perceptions of relationships. Therefore, investigating the self-presentation behaviors and interpersonal relationship satisfaction of high school students on social network sites and relevant psychological factors—can provide an empirical basis for fostering positive social behaviors, establishing healthy relationships, and enhancing individuals’ subjective well-being.

The Positive Prediction of Self-Presentation on Social Network Sites for Relationship Satisfaction.

Interpersonal relationship satisfaction refers to an individual’s cognitive evaluation of their social connections. Each person judges the quality of their relationships with others based on their own standards; fulfillment of these standards results in a sense of satisfaction (Vaughn and Matyastik Baier, 1999). Interpersonal relationship satisfaction is thus an overall evaluation of one’s psychological connections with others based on personal criteria, and it serves as one of the key factors contributing to personality development.

Self-presentation refers to any behavior aimed at conveying an impression of oneself to others (Benoit, 1997). It is a subclass of impression management through which individuals establish their identity and social roles by means of interactive feedback (Brown, 2007). Self-presentation involves not only the management of impressions conveyed to others but also the individual’s efforts to regulate their own self-image (Greenwald and Breckler, 1985). Interpersonal interactions on social network sites can serve as a complementary form to face-to-face social interactions, and individuals have substantial control over how and to what extent they present themselves, making social network sites an ideal platform for self-presentation (Liu et al., 2020). Self-presentation on social network sites refers to users communicating their thoughts, experiences, needs, opinions, knowledge, lifestyles, behavioral patterns, and aspects of “the self” (Baz, 2018). Researchers have categorized self-presentation on these platforms into two types: positive self-presentation, which involves selectively sharing positive personal information, and honest self-presentation, which involves disclosing genuine personal information without concealment (Kim and Lee, 2011). Self-presentation on social network sites represents a digital extension of traditional self-presentation, serving as a secondary platform reflecting real-world interpersonal relationships. Individuals can choose between positive or honest self-presentation based on their self-expectations (Wang et al., 2020). Furthermore, honest self-presentation on social network sites can enhance individuals’ perceived social support and increase interpersonal closeness (Huang, 2016). Social network sites provide new opportunities for self-presentation, as well as for establishing and maintaining relationships. Honest self-presentation on social media facilitates positive interpersonal interactions with others (Zhao, 2017). Therefore, the sincerity and trust conveyed through honest self-presentation are more conducive to positive social interactions among adolescents, thereby contributing to higher evaluations of interpersonal relationship satisfaction. Individuals who engage in positive self-presentation on social network sites focus on highlighting favorable aspects of themselves, aiming to reduce or eliminate the negative impact of events or information that may threaten their self-concept (Lee et al., 2011). Research by Li et al. (2018) indicates that positive self-presentation on social network sites can help individuals receive more positive feedback from others, increase opportunities for interaction, further narrow social distance, and reduce feelings of loneliness. According to Pang (2018) when people disclose their inner thoughts or emotional states to others in social network sites it not only helps foster friendship maintenance but also leads to greater life satisfaction. Thus, positive self-presentation may convey a more optimistic attitude toward life to other users on social network sites, elicit more prosocial behaviors from them, and lead to more positive evaluations of interpersonal relationships.

In summary, adolescents can consciously choose whether to present an honest or a positive self-image on social network sites. The different self-images they project online often elicit distinct types of interactions and feedback from other users, which may in turn shape their perceptions of online interpersonal relationships. If they present a self-image that is both honest and positive, they are more likely to gain recognition and support from others by conveying optimistic and proactive qualities. This also creates opportunities for higher-quality interactions, enhances their sense of belonging, and leads to more favorable evaluations of relationship satisfaction. Therefore, this study proposes Hypothesis 1: Both positive self-presentation and self-presentation on social network sites can positively predict interpersonal relationship satisfaction among high school students.

The Mediating Role of Self-Esteem Between Social Network Site Self-Presentation and Interpersonal Relationship Satisfaction.

Self-esteem refers to an individual’s subjective evaluation of their own worth as a person (MacDonald and Leary, 2012). Research by Gonzales and Hancock (2011) indicated that self-presentation on Facebook can predict an increase in users’ self-esteem. On online social platforms, taking and sharing selfies is a common form of self-presentation. Users express themselves by posting photos visible to friends or strangers on these platforms. Through social comparison on social platforms, the feedback individuals receive plays a crucial role in the recognition of self-worth and the formation of self-esteem (Wei, 1998). Specific forms of feedback, such as the number of “likes” on a user’s selfies, can encourage further selfie-posting behaviors to enhance their self-esteem. Research conducted by Niu et al. (2015) among university students showed that both positive self-presentation and honest self-presentation on social network sites are significantly positively correlated with self-esteem. Liu (2021) found in a study of adolescents that honest self-presentation on social network sites significantly and positively predicts self-esteem. The development of self-esteem helps individuals accumulate psychological resources, experience more positive emotions, and exhibit higher life satisfaction (Jia et al., 2018). The positive role of self-esteem in interpersonal relationships has also been validated in online contexts. Self-esteem, as a central mechanism in interpersonal relationships, exerts a profound influence on their establishment and maintenance (Wu, 2025). People with normal to high self-esteem were more inclined to use social networks via smartphones, maintained a larger number of friends and followers on these platforms, and demonstrated the potential to foster friendship development under certain circumstances (Vagka et al., 2024). According to Consequently, individuals with higher levels of self-esteem are more proactive in forming friendships and maintaining better social relationships. Therefore, the interactions and feedback resulting from high school students’ self-presentation behaviors on online social platforms may influence their self-esteem, which in turn affects the quality of their interpersonal relationships and their level of relationship satisfaction. This study further explores the role of self-esteem in the relationship between social network site use and interpersonal relationship satisfaction among high school students, proposing Hypothesis 2: Self-esteem mediates the relationship between both positive and honest self-presentation on social network sites and interpersonal relationship satisfaction among high school students.

The Mediating Role of Self-Identity Between Self-Presentation on Social Network Sites and Interpersonal Relationship Satisfaction.

Self-identity refers to the process of discovering one’s true self and understanding who one truly is—a concept introduced by the American psychologist Erik Erikson in the 1960s (Gerrig et al., 2015). Chinese scholar Huang (2002) posited that self-identity constitutes an individual’s answers to all questions related to the self. According to Erikson’s theory of psychosocial development, adolescence is a critical period for the formation of self-identity. During this stage, adolescents need to develop an integrated and coherent personal framework and a set of effective behavioral patterns to guide and regulate their actions (Yu and Luo, 2016). Adolescents in this phase become particularly concerned with how they are perceived by others and their emotional position within social groups. The online self-expression of adolescents can reflect their thoughts, opinions, fantasies, and imaginations as represented in digital environments. The online self often encompasses both personal and social dimensions, and establishing a stable, unified self-identity is an essential developmental task for adolescents (Subrahmanyam and Šmahel, 2011). For emerging adulthood, honest online self-presentation helps strengthen self-concept clarity (Wong and Hamza, 2025). Among middle school students, self-identity can directly influence peer interactions (Gong, 2024). Individuals with more developed self-identity tend to exhibit greater self-awareness and self-evaluation, are more skilled at handling peer relationships, and experience higher levels of social acceptance. In summary, positive feedback received from presenting an honest and positive self on social network sites can help individuals integrate external perspectives into their self-concept, thereby promoting the development of self-identity. When individuals form a stable self-concept, that is, when their self-identity is well-developed, adolescence are more capable of expressing themselves honestly and positively, managing interpersonal relationships more effectively, and experiencing greater satisfaction in their social interactions. This study proposes Hypothesis 3: Self-identity mediates the relationship between both honestly and positive self-presentation on social network sites and interpersonal relationship satisfaction among high school students.

The Chain Mediating Role of Self-Esteem and Self-Identity Between Self-Presentation on Social Network Sites and Interpersonal Relationship Satisfaction.

Self-esteem and self-identity may function as mediating variables between online self-presentation and interpersonal relationship satisfaction among high school students. When multiple mediators exist between an independent variable and a dependent variable, these variables may operate through parallel multiple mediation, chain multiple mediation, or composite multiple mediation (Liu and Ling, 2009). According to the core-periphery theory of self-esteem, self-esteem generally originates from intimate interactions between infants and their primary caregivers, forming a sense of self-worth and security. The affective theory of self-esteem development proposes that self-esteem should form during early individual experiences, suggesting that the construction of self-esteem is based on a global self-feeling developed from these early experiences (Chen and Wang, 2007), with greater emphasis on one’s own judgment and understanding of self-worth. According to Erikson’s theory of psychosocial development, the primary developmental task for adolescents aged 12 to 18 is resolving the conflict between identity formation and role confusion. The construction of self-identity constitutes a crucial developmental challenge during this stage (Yu and Luo, 2016). Throughout the process of identity exploration, individuals gradually form a relatively stable understanding of fundamental issues related to the self and personal development. Therefore, from a developmental timeline perspective, self-esteem emerges at an earlier stage of life compared to self-identity. The content encompassed by self-identity builds upon the foundation of self-esteem, self-identity is closely intertwined with social identity, which refers to those socially derived aspects of an individual’s self-concept (Branje et al., 2021). Therefore, there may be a sequential relationship between self-esteem and self-identity, with self-identity likely being a more proximal influencing factor on interpersonal relationship satisfaction. Based on the above, this study proposes Hypothesis 4: Self-esteem and self-identity play a chain-mediating role between social network site self-presentation and interpersonal relationship satisfaction among high school students.

## 2 Methods

To understand the underlying psychological factors of self-presentation behaviors on social network sites among contemporary high school students and to test the proposed chain mediation model, this study employed a cross-sectional design. The independent variable is self-presentation on social network sites, the dependent variable is interpersonal relationship satisfaction, and self-esteem and self-identity serve as the mediating variables.

### 2.1 Participants

This survey was conducted between March and April 2023. Participants were recruited from six secondary schools in Xiamen, China, via a convenience sampling method. Data were collected in a group-testing setting. A total of 750 questionnaires were distributed. After excluding invalid responses, 711 valid questionnaires were retained (94.8% response rate). The final sample comprised 338 male students (47.54%) and 373 female students (52.46%), with participants’ ages ranging from 14 to 17 years (M = 15.76, SD = 0.55).

### 2.2 Measures

#### 2.2.1 Questionnaire of Self-Presentation in SNS

The study employed: The Positive Self-Presentation Questionnaire (6 items; Kim and Lee, 2011; revised by Niu et al., 2015), measuring selective display of positive attributes on social networks (e.g., “Regardless of my true feelings, I only post content that makes me appear happy”). The Honest Self-Presentation Questionnaire (4 items; Kim and Lee, 2011; adapted by Niu et al., 2015), assessing truthful disclosure of thoughts and emotions online (e.g., “I share photos that show my genuine self”). Both questionnaires employed a 7-point Likert scale (1 = strongly disagree to 7 = strongly agree). The scales have demonstrated good reliability and validity in prior studies (Niu et al., 2015; Liu et al., 2017). In the current study, the internal consistency coefficients (Cronbach’s *α*) were 0.63 and 0.77 for the respective scales.

#### 2.2.2 Self-Esteem Scale

The study employed the Rosenberg Self-Esteem Scale (SES), originally developed by Rosenberg (1965) and translated/adapted into Chinese by Wang et al. (1999). This 10-item unidimensional measure uses a 4-point Likert scale (1 = strongly disagree to 4 = strongly agree). The total score ranges from 10 to 40, with scores below 25 indicating low self-esteem and scores above 33 reflecting high self-esteem. Higher scores represent greater self-esteem levels. In the current study, the scale demonstrated excellent internal consistency (*α* = 0.91).

#### 2.2.3 Self-Identity Questionnaire

The study employed the Self-Identity Questionnaire developed by Sun (2005), based on Huang Xiting’s in-depth interviews centering on “Who am I?” and “Where am I going?”, integrated with Erikson’s classical identity theory. The 36-item scale comprises six dimensions: Time Diffusion (7 items), Self-Consciousness (6 items), Energy Dispersion (5 items), Identity Diffusion (7 items), Authority Confusion (4 items), and Directional Loss (7 items). Responses were recorded on a 5-point Likert scale (1 = strongly disagree to 5 = strongly agree). The full scale demonstrated excellent internal consistency (*α* = 0.90), with subscale α coefficients ranging from 0.63 to 0.78.

#### 2.2.4 Interpersonal Satisfaction Questionnaire

The study utilized the Interpersonal Relationship Satisfaction Scale developed by Zhao (2006), which conceptualizes individuals’ perceptions of relationship quality as a bipolar continuum from satisfaction to dissatisfaction. This instrument comprises two subscales: the Satisfaction Subscale and Dissatisfaction Subscale. The current research employed the 22-item Satisfaction Subscale, measuring four dimensions: Mutual Benefit and Support (7 items), Social Competence (6 items), Similarity and Compatibility (4 items), and Moral Integrity (5 items). Responses were recorded on a 6-point Likert scale (1 = strongly disagree to 6 = strongly agree). The scale demonstrated excellent internal consistency (α = 0.96), with subscale α coefficients ranging from 0.85 to 0.93.

### 2.3 Statistical analyses

The study first utilized Harman’s single-factor test to evaluate potential common method bias. Subsequently, correlation and descriptive analyses were conducted to examine the relationships among the study variables. Finally, validation analyses, including serial mediation tests, were performed to assess the proposed model. All statistical analyses were carried out using SPSS version 20 for Windows.

## 3 Results

### 3.1 Common method bias test

This study employed Harman’s single-factor test (Zhou and Long, 2004) to statistically assess common method bias. An exploratory factor analysis (EFA) of all items using SPSS 20.0 revealed that the first common factor accounted for 24.35% of the variance, significantly below the recommended threshold of 40%, indicating no severe common method bias in the data.

### 3.2 Correlation and descriptive analyses

Demographic variables, including gender, age, place of origin, only-child status, as well as mean scores for honest self-presentation on social network sites, positive self-presentation, self-esteem, self-identity, and interpersonal relationship satisfaction were included in the correlation analysis. The means and correlation matrix for all variables and dimensions are presented in Table 1. As shown in Table 1, honest self-presentation was significantly positively correlated with self-esteem, self-identity, and interpersonal relationship satisfaction (*p*s < 0.001). Positive self-presentation was significantly positively correlated with interpersonal relationship satisfaction (*p* < 0.01). Self-esteem was significantly positively correlated with both self-identity and interpersonal relationship satisfaction (*p*s < 0.001). Self-identity was also significantly positively correlated with interpersonal relationship satisfaction (*p* < 0.001).

**Table 1:** Descriptive statistics and correlations among variables.

Variables	M	SD	1	2	3	4	5	6	7
1. Gender	–	–	1						
2. Place of origin	–	–	0.02	1					
3. Age	15.76	0.55	−0.07	−0.08^*^	1				
4. Honest self-presentation	4.29	1.23	0.13^**^	0.06	−0.05	1			
5. Positive self-presentation	3.91	1.21	0.08^*^	−0.02	0.05	0.02	1		
6. Self-esteem	3.66	0.77	0.02	−0.01	0.07	0.25^***^	0.05	1	
7. Self-identity	3.22	0.56	0.00	0.00	0.03	0.24^***^	−0.01	0.67^***^	1
8. Interpersonal relationship satisfaction	4.53	0.84	0.07	0.01	0.05	0.30^***^	0.12^**^	0.44^***^	0.46^***^

*N* = 711. Only-child status was coded as a dummy variable (0 = non-only child, 1 = only child). ^*^ *p* < 0.05, ^**^ *p* < 0.01, ^***^ *p* < 0.001. The same applies hereafter.

None of the demographic variables showed a significant correlation with the dependent variable, interpersonal relationship satisfaction (*p*s > 0.05). Therefore, demographic variables were not included as control variables in subsequent analyses.

### 3.3 Analysis of the chain mediation model

Based on the correlation matrix presented in Table 1, a mediation analysis was conducted to examine the roles of self-esteem and self-identity as mediators between honest self-presentation (the independent variable) and interpersonal relationship satisfaction (the dependent variable). The results indicated that honest self-presentation significantly predicted self-esteem (*β* = 0.25, *p* < 0.001). Furthermore, both honest self-presentation (*β* = 0.08, *p* < 0.05) and self-esteem (*β* = 0.65, *p* < 0.001) significantly predicted self-identity. Finally, honest self-presentation (*β* = 0.18, *p* < 0.001), self-esteem (*β* = 0.21, *p* < 0.001), and self-identity (*β* = 0.27, *p* < 0.001) all significantly predicted interpersonal relationship satisfaction. The full results of the regression analysis are presented in Table 2.

**Table 2:** Chain mediation regression analysis of honest self-presentation, self-esteem, self-identity, and interpersonal relationship satisfaction.

Variables	Self-esteem			Self-identity			Interpersonal relationship satisfaction		
	*β*	*SE*	*t*	*β*	*SE*	*t*	*β*	*SE*	*t*
Honest self-presentation	0.25	0.02	7.00^***^	0.08	0.01	2.66^**^	0.18	0.02	5.36^***^
Self-esteem				0.65	0.02	22.72^***^	0.21	0.05	4.81^***^
Self-identity							0.27	0.06	6.26^***^
*R* ^2^	0.06			0.46			0.27		
*F*	49.04^***^			296.15^***^			87.27^***^		

All variables in the model have been standardized. ^*^ *p* < 0.05, ^**^ *p* < 0.01, ^***^ *p* < 0.001. The same applies hereafter.

A chain mediation analysis was conducted using Model 6 of the PROCESS macro for SPSS (Version 3.3). The indirect effects were tested using 5,000 bootstrap samples with 95% bias-corrected confidence intervals. As shown in Table 3, this total mediating effect consists of three specific indirect effects: First, the path through self-esteem (honest self-presentation → self-esteem →interpersonal relationship satisfaction) had an indirect effect of 0.05, accounting for 16.67% of the total effect, with a 95% confidence interval of [0.03, 0.09], indicating significant mediation. Second, the path through self-identity only (honest self-presentation→ self-identity→ interpersonal relationship satisfaction) produced a significant indirect effect of 0.02 (6.67% of the total effect), with a 95% CI of [0.003, 0.04]. Third, the chain mediation path through self-esteem and self-identity (honest self-presentation → self-esteem → self-identity → interpersonal relationship satisfaction) also showed a significant indirect effect of 0.05 (16.67% of the total effect), with a 95% CI of [0.03, 0.07].

**Table 3:** Mediation analysis of self-esteem and self-identity in the relationship between honest self-presentation and interpersonal relationship satisfaction.

Effect types	*β*	*SE*	95% Confidence interval		Effect size
			Lower limit	Upper limit	
Direct effect	0.18	0.03	0.11	0.24	60.00
The honest self-presentation → self-esteem → interpersonal relationship satisfaction	0.05	0.02	0.03	0.09	16.67
The honest self-presentation → self-identity →interpersonal relationship satisfaction	0.02	0.01	0.003	0.04	6.67
The honest self-presentation → self-esteem → self-identity →interpersonal relationship satisfaction	0.05	0.01	0.03	0.07	16.67
Total indirect effect	0.12	0.02	0.08	0.16	40.00
Total effect	0.30	0.04	0.23	0.37	100.00

**Figure 1:**
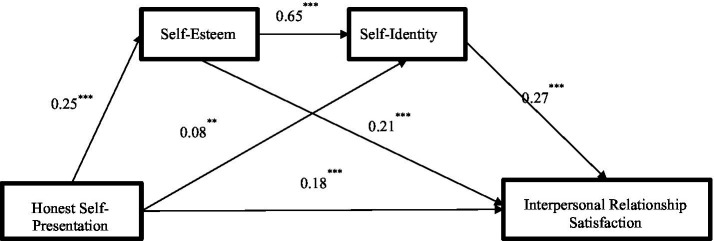
Path diagram of the effect of honest self-presentation on interpersonal relationship satisfaction.

As shown in Figure 1, honest self-presentation significantly and positively predicted interpersonal relationship satisfaction (*β* = 0.18, *p* < 0.001). Honest self-presentation also significantly predicted self-esteem (*β* = 0.25, *p* < 0.001), self-esteem significantly predicted self-identity (*β* = 0.65, *p* < 0.001), and self-identity significantly predicted interpersonal relationship satisfaction (*β* = 0.27, *p* < 0.001). These results indicate that self-esteem and self-identity play a chain mediating role in the relationship between honest self-presentation and interpersonal relationship satisfaction.

## 4 Discussion

### 4.1 The influence of honest and positive self-presentation on social network sites on interpersonal relationship satisfaction among high school student

The results of this study indicate that both honest and positive self-presentation on social network sites significantly and positively predict interpersonal relationship satisfaction among high school students. Social penetration theory (Altman and Taylor, 1973) posits that revealing a comprehensive and honest self to others is central to the development of interpersonal relationships, suggesting that expressing one’s true self facilitates the establishment of social bonds. When interacting with netizens, sharing a positive and optimistic attitude toward life allows individuals to experience others’ satisfaction with them, as well as feel the support from others (Gable et al., 2003). This finding also aligns with the motivation theory of self-presentation (Tedeschi et al., 1971). Individuals who present their honest and positive selves on social network sites tend to have a better understanding of the expectations and rules of interaction in the online social world, as well as a clearer awareness of their own and others’ needs on these platforms. In their pursuit of more approval and recognition, they are more likely to present content that aligns with societal expectations. When high school students share more honest personal information on social network sites, the sincerity and genuineness they convey can lead to more positive feedback in interpersonal interactions, resulting in higher evaluations of relationship satisfaction. Additionally, when high school students convey optimistic and positive attitudes online, it can enhance their social attractiveness to others and foster more prosocial behaviors. These positive experiences become important factors influencing high school students to rate their interpersonal relationship satisfaction more highly.

### 4.2 Mediating roles of self-esteem and self-identity

The results of this study indicate that self-esteem mediates the relationship between honest self-presentation on social network sites and interpersonal relationship satisfaction among high school students. Furthermore, self-identity also serves as a mediator in this relationship. Finally, self-esteem and self-identity together play a chain-mediating role between honest self-presentation on social network sites and interpersonal relationship satisfaction.

The mediating role of self-esteem between honest self-presentation on social network sites and interpersonal relationship satisfaction among high school students can be explained from several perspectives. Users often regard social network sites as platforms for self-verification and gaining social recognition (Mehdizadeh, 2010). They may adopt various self-presentation strategies to enhance how others perceive them in the online environment, thereby improving their self-evaluation. Yang and Bradford Brown (2016) found that self-presentation on Facebook was positively correlated with the feedback received, which in turn influenced individuals’ self-esteem. Self-esteem emphasizes an individual’s evaluation of their own valuable qualities. Students who present their honest selves on social network sites are more likely to accept their true identity and recognize their self-worth. The genuine qualities they display are more inclined to receive positive feedback from others, leading to higher satisfaction in their interpersonal relationships.

The mediating role of self-identity between honest self-presentation on social network sites and interpersonal relationship satisfaction among high school students can be understood through several perspectives. The development of online social platforms has provided a stage for the construction of personal social identity (Jin, 2024). Adolescents can more freely explore multiple aspects of themselves in the virtual world, which facilitates the development of self-identity (Yao et al., 2014) and promotes a more integrated and coherent understanding of the self, including how they perceive and evaluate their interpersonal relationships. Social network sites have altered how teenagers present themselves online, allowing them to enhance social skills, expand their social networks, and obtain social support at relatively low emotional and transactional costs, thereby increasing their sense of life satisfaction (Ding, 2017). These experiences help individuals form more comprehensive and positive self-evaluations, as well as greater satisfaction with their social groups and relationships.

The present study also found that honest self-presentation on social network sites influences high school students’ interpersonal relationship satisfaction through the chain-mediating path of self-esteem and self-identity. The development of self-esteem and self-identity among adolescents follows a sequential pattern, wherein the development of self-identity is influenced by prior development of self-esteem. This result is consistent with the study by Zhang (2016), which indicated a close correlation between self-esteem and self-identity. Specifically, positive and stable self-esteem facilitates healthy self-confirmation, thereby contributing to the formation and development of a coherent self-identity. High school students who tend to present their honest selves more on social network sites are more likely to accept their true selves and recognize their self-worth. Building on a foundation of developed self-esteem, they form more comprehensive and positive perceptions of self-related issues, which in turn leads to higher satisfaction in interpersonal relationships.

Finally, some individuals tend to present a more ideal self on social network sites (Vazire and Gosling, 2004). Although this identity may not fully reflect their current actual self, feedback from other users can help them recognize previously unnoticed strengths and resources, leading to the realization that they are better than they have thought. By reinforcing their self-worth and broadening their self-awareness, they gradually align their actual self with their ideal self. When individuals accomplish this developmental process, they are likely to engage in more positive interpersonal behaviors and experience higher satisfaction in their relationships.

However, it is noteworthy that in this study, positive self-presentation was not significantly correlated with self-esteem or self-identity. For high school students, when a discrepancy exists between the actual self and the ideal self, it may trigger negative self-evaluations and raise doubts about self-worth. These adolescents may post exaggerated or idealized images of themselves online. If such presentations are perceived as artificial or insincere, they may become a source of negative self-assessment, further reinforcing the individual’s negative self-views and contributing to low self-esteem (Liu et al., 2021). This conclusion has also been empirically supported by studies conducted in Western contexts. According to a research in McMaster University in Canada, some students craft idealized online identities to meet social expectations, driven by a need for approval and fear of criticism. This behavior increases self-consciousness and distorts self-image (Hunking et al., 2024). Furthermore, exposure to curated peer content worsens feelings of inadequacy and perceived competition, negatively impacting mental health and social relationships.

Excessively exaggerated or idealized online self-presentations may lead individuals to constantly compare their digital self with their real-life identity. This comparison can amplify the perceived gap between the two selves, and such self-discrepancy may adversely impact well-being (Yu and Kim, 2020). These perspectives warrant further investigation in future research.

## 5 Implications and limitations

This study examined the impact of high school students’ self-presentation on social network sites on their interpersonal relationship satisfaction, and further explored the underlying psychological mechanisms, thereby enriching previous research and providing insights for designing mental health education content for adolescents. In educational practice, we should foster adolescents’ ability to differentiate between their online and offline personas and pursue a seamless integration of the two. Concurrently, digital literacy must be established as a key element of mental health education curricula. From an developmental perspective, interpersonal relationships constitute a crucial aspect of high school students’ lives. This study suggests that efforts to enhance adolescents’ self-esteem and facilitate the development of self-identity can be effective ways to support their psychosocial growth. By fostering alignment between their actual and ideal selves, and deepening their self-awareness and sense of self-worth, educators and practitioners can help promote positive social experiences and enhance overall well-being among high school students. Additionally, effective online interventions should equip adolescents with the skills to discern manipulated online content. This can shield them from the anxiety of social comparison, redirect their focus to offline activities, and empower them to build fulfilling relationships through positive, real-world social interactions.

This study has several limitations. First, the cross-sectional design does not capture developmental changes in adolescents’ self- presentation on social network sites, self-esteem, self-identity, and interpersonal relationship satisfaction, nor can it establish causal inferences. Second, In this study, the reliability coefficient for positive self-presentation was 0.63, indicating acceptable but not high reliability. This may be attributed to potential comprehension discrepancies influenced by cultural differences. For instance, within Chinese culture, which highly values modesty as a virtue—individuals may be less accustomed to openly displaying their positive aspects in public contexts. Subsequent revisions of the scale must adequately account for the differences between Chinese and Western contexts. Third the sample was limited to high school students, which constrains the generalizability of the findings. Since high school students are in the middle to late stages of adolescence, their self-perception and level of self-esteem are relatively more stable compared to younger adolescents, which may influence their social media behaviors and perceptions of relationships in distinct ways. Fourth, the study did not analyze the extent or content of self-presentation. Gender differences may exist in the type of content shared on different platforms; for example, female students might be more inclined to share selfies, while male students may more frequently post gaming achievements. Moreover, with the evolution of social network technologies, self-presentation may increasingly involve virtual avatars or cartoon representations, making the assessment of self-presentation strategies more complex. Finally, while existing international research on online self-presentation often focuses on platforms such as Instagram and Facebook, most domestic studies do not specify which social network sites were examined. Different high school students may prefer different platforms, and this study did not investigate which sites the participants used. Adolescents may present different facets of themselves depending on a platform’s affordances and privacy features, which could lead to varying types of online feedback and differentially influence their self-perception, self-esteem, and relationship satisfaction.

## 6 Conclusion

There were significant positive correlations among honest self-presentation on social network sites, self-esteem, self-identity, and interpersonal relationship satisfaction among high school students. Furthermore, honest self-presentation on social network sites influenced interpersonal relationship satisfaction through the following pathways: a direct predictive effect, the mediating role of self-esteem, and the chain mediating effect of self-esteem and self-identity.

## Data Availability

The raw data supporting the conclusions of this article will be made available by the authors, without undue reservation.
